# Prognostic value of vascular endothelial growth factor (VEGF) in head and neck squamous cell carcinomas

**DOI:** 10.1054/bjoc.2000.1357

**Published:** 2000-08-17

**Authors:** H Mineta, K Miura, T Ogino, S Takebayashi, K Misawa, Y Ueda, I Suzuki, M Dictor, Å Borg, J Wennerberg

**Affiliations:** Department of Otolaryngology, Division of Pathology, Hamamatsu University School of Medicine, Hamamatsu, 431–3192, Japan; Departments of Pathology, Oncology, Otolaryngology/Head and Neck Surgery, Lund University, Lund, 221–85, Sweden

**Keywords:** vascular endothelial growth factor, Western blot, head and neck carcinomas, prognostic indicator

## Abstract

Vascular endothelial growth factor (VEGF) has been identified as the substance that increases the permeability and proliferation of vascular endothelial cells. We examined the clinical significance of VEGF expression in 60 head and neck squamous cell carcinomas using the methods of Western blot, immunohistochemistry, and reverse transcriptase-polymerase chain reaction (RT-PCR), comparatively, and analysed the relationship between VEGF status in Western blot and tumour size, lymph-node status, histologic grade and disease-free survival (DFS) rate. Western blot analysis revealed high VEGF expressors (tumour/normal tissue density ≥ 3-fold) in 26 patients (43%) and low VEGF expressors (< 3-fold) in 34 patients (57%). The results of the Western blot analysis correlated significantly with those of the RT-PCR (*P*= 0.00007) or immunohistochemistry (*P*= 0.00006). High VEGF expressors are associated with the progression of lymph-node spread (*P*= 0.0009), which are correlated with poor DFS. The 2-year DFS rate of high VEGF expressors (30%) was significantly lower than that of low VEGF expressors (78%) (*P*= 0.0008). Multivariate analysis showed VEGF expression and stage were independent predictors for the DFS (*P*= 0.045 and 0.041, respectively). VEGF expression may play an important role in progression of HNSCC. © 2000 Cancer Research Campaign


*British Journal of Cancer* (2000) **83**(6), 775–781
© 2000 Cancer Research Campaign
doi: 10.1054/ bjoc.2000.1357, available online at http://www.idealibrary.com on IDEAL®

### Prognostic value of vascular endothelial growth factor (VEGF) in head and neck squamous cell carcinomas

**H Mineta[1], K Miura[2], T Ogino[1], S Takebayashi[1], K Misawa[1], Y Ueda[1], I Suzuki[1], M Dictor[3], Å Borg[4] and J Wennerberg[5]**

[1]Department of Otolaryngology and [2]Division of Pathology, Hamamatsu University School of Medicine, 431–3192 Hamamatsu, Japan; Departments of [3]Pathology, [4]Oncology and [5]Otolaryngology/Head and Neck Surgery, Lund University, 221–85 Lund, Sweden

**Summary** Vascular endothelial growth factor (VEGF) has been identified as the substance that increases the permeability and proliferation of vascular endothelial cells. We examined the clinical significance of VEGF expression in 60 head and neck squamous cell carcinomas using the methods of Western blot, immunohistochemistry, and reverse transcriptase-polymerase chain reaction (RT-PCR), comparatively, and analysed the relationship between VEGF status in Western blot and tumour size, lymph-node status, histologic grade and disease-free survival (DFS) rate. Western blot analysis revealed high VEGF expressors (tumour/normal tissue density ≥ 3-fold) in 26 patients (43%) and low VEGF expressors (< 3-fold) in 34 patients (57%). The results of the Western blot analysis correlated significantly with those of the RT-PCR ($P$ = 0.00007) or immunohistochemistry ($P$ = 0.00006). High VEGF expressors are associated with the progression of lymph-node spread ($P$ = 0.0009), which are correlated with poor DFS. The 2-year DFS rate of high VEGF expressors (30%) was significantly lower than that of low VEGF expressors (78%) ($P$ = 0.0008). Multivariate analysis showed VEGF expression and stage were independent predictors for the DFS ($P$ = 0.045 and 0.041, respectively). VEGF expression may play an important role in progression of HNSCC. © 2000 Cancer Research Campaign

**Keywords**: vascular endothelial growth factor; Western blot; head and neck carcinomas; prognostic indicator

Angiogenesis is a rare event and tightly controlled under normal physiological conditions, because normal cells secrete only low levels of positive regulators and high levels of negative regulators of angiogenesis (Bouck et al, 1996). Angiogenesis plays an important role not only in wound healing, but also in cancer development. Recently, the mechanism of cancer progression or metastasis has been shown to be associated with angiogenesis (Folkman et al, 1989; Folkman, 1995; Holmgren et al, 1995). The onset of angiogenesis is believed to be an early event in carcinogenesis and this process may facilitate cancer progression and metastasis (Folkman et al, 1989). Vascular endothelial growth factor (VEGF) has been identified as one of the factors that increases the permeability (Senger et al, 1983; Ferrara and Henzel, 1989), and it is known to be the same as vascular permeabilty factor (VMP) (Keck et al, 1989; Leung et al, 1989; Ferrara et al, 1991*a*; 1992). Four different isoforms with $VEGF_{121}$, $VEGF_{165}$, $VEGF_{189}$ and $VEGF_{206}$ were identified by the alternating splicing of mRNA (Ferrara et al, 1991*b*). Although VEGF contains a signal peptide to direct secretion, only the two shorter forms ($VEGF_{121}$ and $VEGF_{165}$) are efficiently secreted. The two high molecular weight forms ($VEGF_{189}$ and $VEGF_{206}$) seem to be mostly cell associated (Ferrara et al, 1991*b*). VEGF secreted from tumour cells causes the proliferation of surrounding endothelial cells through a paracrine system (Brown et al, 1988) and associates closely with dense vessels in tumour tissue. Tumour cells can easily invade neovascular vessels because the basement membranes of these vessels are immature.

*Received 14 March 2000*
*Accepted 17 April 2000*

*Correspondence to*: H Mineta

VEGF expression was found in a wide variety of human carcinomas including brain (Takano et al, 1996), breast (Toi et al, 1996; Yoshiji et al, 1996; Linderholm et al, 1998; Scott et al, 1998), lung (Fontanini et al, 1997; 1999; Shibusa et al, 1998), oesophageal (Inoue et al, 1997; Shimada et al, 1999), gastric (Maeda et al, 1995; 1999; Saito et al, 1999), colorectal (Takahashi et al, 1995; 1997; Ishigami et al, 1998; Kumar et al, 1998), pancreatic (Itakura et al, 1997; Ikeda et al, 1999), hepatocellular (Suzuki et al, 1996), renal and bladder (Brown et al, 1993; Takahashi et al, 1994; O'Brien et al, 1995; O'Byrne et al, 1999), ovarian (Olson et al, 1994; Boocock et al, 1995; Abu-Jawdeh et al, 1996; Paley et al, 1997) and head and neck carcinomas (Eisma et al, 1997; Moriyama et al, 1997; Salven et al, 1997; Maeda et al, 1998; Burian et al, 1999; Neuchrist et al, 1999; Sauter et al, 1999), and was suggested to play an important role in angiogenesis. VEGF expression was reported to correlate with clinical parameters including tumour size (Itakura et al, 1997; Kumar et al, 1998), lymph-node metastasis (Maeda et al, 1995; Moriyama et al, 1997; Kumar et al, 1998; Sauter et al, 1999), and prognosis (Maeda et al, 1995; Fontanini et al, 1997; Inoue et al, 1997; Linderholm et al, 1998; Saito et al, 1999, and so on), however, this relationship has not been consistent. Although all these studies were mostly analysed only by immunohistochemistry, there have been no reports of this relationship using Western blot, immunohistochemistry and reverse transcriptase-polymerase chain reaction (RT-PCR), comparatively, in head and neck squamous cell carcinomas (HNSCCs). The correlation between VEGF expression and subsites of HNSCCs remains unknown.

The purpose of this study is to examine the level of VEGF expression by Western blot in HNSCCs, and to confirm the precise location of the cells expressing VEGF by immunohistochemistry; to examine the form of VEGF mRNA by RT-PCR; and to examine whether VEGF expression is associated with clinicopathological characteristics and prognosis.

The data presented show that VEGF plays an important role in lymph-node status and outcome in HNSCCs.

### METHODS

### Patients and materials

Sixty patients with HNSCCs without distant metastasis were analysed. All patients were treated at the Department of Otolaryngology, Hamamatsu University School of Medicine, Hamamatsu, Japan. Clinical information including age, sex, tumour size, lymph-node status, stage grouping, histologic grade, and outcome was obtained from the clinical records. Primary tumour size, lymph-node status, and stage grouping were classified according to the 1997 UICC criteria (Sobin and Witterkind, 1997). Two pieces of specimens were collected from the same patients at surgery, one from tumour tissue and another from the adjacent normal tissue. For confirming the specimen feasibility for analysis, all specimens were divided into three parts for Western blot, RT-PCR and histology.

### Western blot

Thirty μg of the protein extract in a lysis buffer containing 100 mM Tris (pH 7.4), 0.15 M NaCl, and 1% SDS was electrophoresed on a 12.5% SDS /polyacrylamide gel and transferred to a Hybond-PVDF membrane (Amersham, Buckinghamshire, England). Blots were first incubated in a blocking buffer (Dulbecco's PBS buffer containing 3% bovin serum albumin and 3% skimmed milk) for 1 h at room temperature, then the blots were immunoblotted with the anti-human VEGF rabbit polyclonal antibody (A-20: Santa Cruz Biotechnology Inc., Santa Cruz, CA, USA) at a dilution of 1:1500 at 4°C overnight. After rinsing the blots with PBS, they were incubated with horseradish peroxidase conjugated anti-rabbit IgG (Amersham) at a dilution of 1:1000 for 1 h at room temperature. After washing the blots, they were developed with a detection kit (ECL, Amersham). To confirm the result of Western blot using A-20 antibody, we performed it using the other anti-human VEGF antibody (A-147: Santa Cruz Biotechnology Inc.) at a dilution of 1:1000 at 4°C overnight. The optimized bands were analysed by a Model GS-700 Imaging Densitometer (Bio-Rad, Hercules, CA, USA) and Molecular Analyst Software/Macintosh (Bio-Rad). The density of VEGF bands was divided into two groups (the ratio of tumour/normal tissue, low < 3-fold; high ≥ 3-fold) by comparing it with the density of the bands that we extracted from the accompanying normal tissue. Actin (anti-actin antibody: 1–19, Santa Cruz Biotechnology Inc., 1:1000 dilution) levels were analysed as controls for protein loading. Tumours which showed high intensity of VEGF expression for A-20 were considered to overexpress VEGF.

### RT-PCR

Total RNA was extracted using an ISOGEN kit (Nippon Gene, Toyama, Japan) and RT-PCR was carried out using a RT-PCR high kit (TOYOBO, Osaka, Japan), according to the manufacturers' protocols. PCR primers were as follows: forward primer 5′-TCGGGCCTCCGAAACCATGA-3′ and reverse primer 5′-CCTGGTGAGAGATCTGGTTC-3′ (Weindel et al, 1992). These primers can amplify the whole coding region of all known splicing forms of VEGF mRNA. The PCR amplification cycle consisted of denaturation at 95°C for 50 s, annealing at 57°C for 50 s, and extension at 72°C for 70 s. This was done for 35 cycles. PCR products were run on a 6% polyacrylamide gel and visualized by ethidium bromide staining. G3PDH levels were analysed as controls.

### Immunohistochemistry

Five μm sections were dewaxed with xylene, hydrated through graded alcohols, and rehydrated in water. Sections were microwaved in a citrate buffer (pH 6.0) three times for 5 min, and endogenous peroxidase activity was blocked using 0.5% hydrogen peroxide in methanol for 30 min. A 20% goat serum was applied to the sections for 10 min as a blocking reagent to reduce non-specific binding. A 1:1000 dilution of the monoclonal antibody against VEGF protein (R&D, Abingdon, UK) was used. Sections were incubated at 4°C overnight. They were incubated with the biotinylated anti-mouse immunoglobulin rabbit antibody (DAKO, Copenhagen, Denmark) for 30 min, followed by incubation with streptavidin peroxidase reagents (Strept-ABComplex: DAKO) for 30 min. They were treated in diaminobenzidine solution for 5 min, and then counterstained with haematoxylin.

### Immunohistochemical evaluation

Immunohistochemical evaluation was performed by a pathologist in a blind test (without knowledge of the clinical parameters and outcome). At least 20 high-power fields from a single tissue section were chosen at random, and 2000 tumour cells were counted. Positive cells were stained dark brown on the cytoplasm. The expression was considered high intensity if the expression was noted in 10% or more tumour cells and was considered low intensity if the expression was noted in fewer than 10% of the tumour cells.

### Statistical analysis

The association between discrete variables and VEGF expression in western blot was tested by the Fisher's exact probability test or the Mann–Whitney *U* test. Cox's proportional hazards regression analysis that included age, sex, histologic grade, tumour size, lymph-node status, stage grouping and VEGF expression was used to identify the multivariate predictive value of the prognostic factors. Disease-free survival (DFS) curves were constructed using the method of Kaplan–Meier and tested by the log-rank test. A significant difference was identified when the probability was less than 0.05.

### RESULTS

Clinical data of 60 patients with HNSCCs are summarized in Tables 1 and 2. Western blot using two anti-VEGF antibodies (A-20 and A-147) showed similar results. By the Western blot results of A-20, we categorized the HNSCCs into two groups, high VEGF expresser and low VEGF expressor (Table 1, Figure 1A). Western blot analysis revealed high VEGF expression in 26 patients (43%)

 © 2000 Cancer Research Campaign

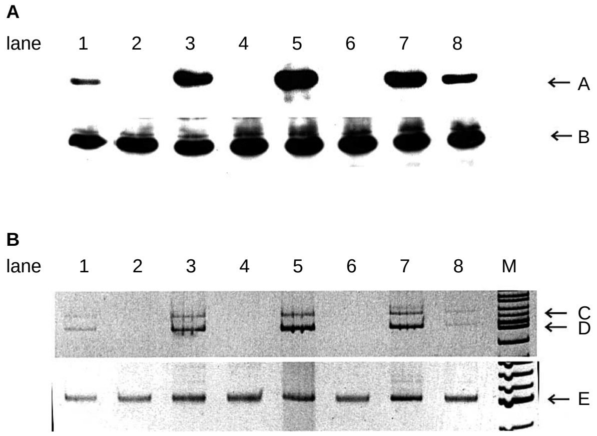

**Figure 1** Vascular endothelial growth factor (VEGF) protein and mRNA analyses. A: 42 kD (VEGF); B: 17 kD (Actin); C: 650 bp (VEGF165); D: 520 bp (VEGF121); E: 450 bp (G3PDH). Lanes 1 and 2: tumour area and normal area in the patient with tongue carcinoma, lanes 3 and 4: tumour area and normal area in the patient with hypopharyngeal carcinoma, lanes 5 and 6: tumour area and normal area in the patient with laryngeal carcinoma, lanes 7 and 8: tumour area and normal area in the patient with hypopharyngeal carcinoma, and lane M: molecular weight marker of RT-PCR. (**A**) VEGF protein was demonstrated by Western blot in lanes 1, 3, 5,7, and 8. The density of lane 7 was 3.2-fold higher than that of lane 8. Actin levels were controlled for protein loading. (**B**) VEGF mRNA was showed by RT-PCR in lanes 1, 3, 5, 7, and 8. The 650 bp and 520 bp encoded VEGF165 and VEGF121, respectively. Two longer forms (VEGF206 and VEGF189) were not detected. G3PDH levels were used as controls

**Table 1** VEGF expression by Western blot and primary site in 60 patients with head and neck squamous cell carcinomas

| Primary site | High VEGF expressors ($n$ = 26) | Low VEGF expressors ($n$ = 34) |
|---|---|---|
| Oral cavity | 9 (45%) | 11 (55%) |
| Larynx | 9 (56%) | 7 (44%) |
| Maxillary sinus | 1 (8%) | 11 (92%) |
| Oropharynx | 5 (71%) | 2 (29%) |
| Hypopharynx | 2 (40%) | 3 (60%) |

(average ± SD, range: 10.02 ± 6.73, 3.1–22.1) and low VEGF expression in 34 patients (57%) (0.99 ± 0.61, 0.01–1.8). High VEGF expressors were found in 71% (5/7) of oropharyngeal carcinoma, 56% (9/16) of laryngeal carcinoma, 45% (9/20) of oral carcinoma, 40% (2/5) of hypopharyngeal carcinoma, and 8% (1/12) of maxillary carcinoma. The incidence of maxillary carcinomas was significantly lower than that of others ($P$ = 0.006).

RT-PCR analysis showed that in 22 patients two forms of transcripts were detected, which encoded for $VEGF_{121}$ and $VEGF_{165}$, and in 38 patients they were not (Figure 1B). The result of RT-PCR was significantly correlated with that of Western blot ($P$ = 0.00007).

Immunohistochemical analysis showed that VEGF positive reactivity was found in 35 patients, and not in 25 patients. The positive cells of expressing VEGF were almost all tumour cells. Tumour infiltrating lymphocytes, endothelial cells, and fibroblasts also showed positive reactivity, although its contribution to the whole tissue was minor (Figure 2). The result of immunohisto-

**Table 2** VEGF expression by Western blot according to clinicopathological characteristics and VEGF RT-PCR and VEGF immunostaining analyses

| Patient and tumour characteristics | High VEGF expressors ($n$ = 26) | Low VEGF expressors ($n$ = 34) | $P$-value |
|---|---|---|---|
| Age (Mean) | 37–90 (63) | 21–79 (60) | NS[a] |
| Sex (Men:Women) | 23:3 | 23:11 | NS[a] |
| Histologic grade | | | |
| Poorly | 3 | 4 | NS[b] |
| Moderately | 8 | 11 | |
| Well | 15 | 19 | |
| Tumour size | | | |
| T1 | 2 | 3 | NS[b] |
| T2 | 11 | 11 | |
| T3 | 7 | 6 | |
| T4 | 6 | 14 | |
| Lymph-node status | | | |
| N0 | 4 | 21 | 0.0009[b] |
| N1 | 7 | 6 | |
| N2 | 10 | 7 | |
| N3 | 5 | 0 | |
| Stage | | | |
| I | 1 | 2 | NS[b] |
| II | 3 | 9 | |
| III | 4 | 5 | |
| IV | 18 | 18 | |
| VEGF RT-PCR | | | |
| Positive | 17 | 5 | 0.00007[a] |
| Negative | 9 | 29 | |
| VEGF immunostaining | | | |
| Positive | 23 | 12 | 0.00006[a] |
| Negative | 3 | 22 | |

[a]Fisher's exact probability test; [b]Mann–Whitney U test; NS = not significant

© 2000 Cancer Research Campaign

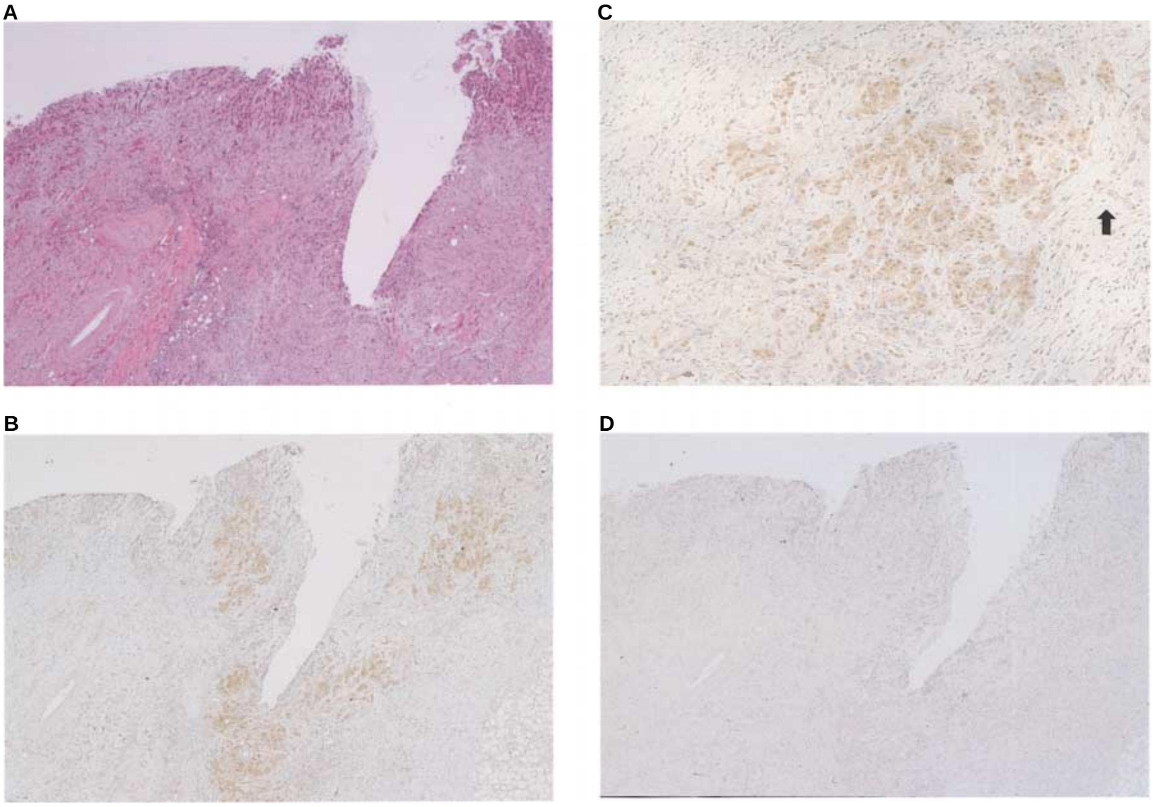

**Figure 2** Haematoxylin-eosin (HE) staining and vascular endothelial growth factor (VEGF) immunostaining of tumour tissue from the patient with tongue carcinoma. (**A**) Well differentiated squamous cell carcinoma of the tongue shows invasive growth (HE staining) (original magnification × 40). (**B**) VEGF immunostaining of tumour tissue in the same lesion as **A** (× 25). The positive reactivity of almost tumour cells and vascular endothelial cells are shown (× 40). (**C**) VEGF immunostaining of tumour tissue in high magnification. The cytoplasm of the tumour cells are stained positive. The fibroblasts, vascular endothelial cells (arrow), and lymphocytes around the tumour are also stained positive (× 100). (**D**) No cells are stained using PBS as the first antibody instead of anti-VEGF antibody (negative control) (× 25)

**Table 3** Risk factors affecting disease-free survival (DFS) rate, determined by Cox's proportional hazards model

| Variable | Relative risk | 95% CI | *P*-value |
|---|---|---|---|
| Sex | | | |
| Men vs Women | 0.885 | 0.324–2.419 | 0.812 |
| Histologic grade | | | |
| Poorly vs others | 0.594 | 0.215–1.644 | 0.316 |
| Tumour size | | | |
| 1,2 vs 3,4 | 0.572 | 0.177–1.843 | 0.349 |
| Lymph-node status | | | |
| (–) vs (+) | 0.719 | 0.274–1.884 | 0.502 |
| Stage | | | |
| 1,2 vs 3,4 | 8.425 | 1.092–8.086 | 0.041 |
| VEGF expression | | | |
| Negative vs Positive | 0.424 | 0.184–0.980 | 0.045 |

chemistry was significantly correlated with that of Western blot ($P = 0.00006$).

Table 2 shows the clinical differences between high VEGF expressors and low VEGF expressors. The VEGF expression was significantly correlated with lymph-node status ($P = 0.0009$), but not with age, sex, histologic grade, tumour size, and stage grouping. High VEGF expressors displayed more aggressive lymph-node metastasis.

Multivariate analysis using Cox's hazard model revealed that VEGF expression and stage grouping were independent predictors for the DFS ($P = 0.045$ and 0.041, respectively). However, sex, histologic grade, tumour size, and lymph-node status were not (Table 3). Kaplan–Meier curve (Figure 3) demonstrated that the 2-year DFS rate of high VEGF expressors (30%) was significantly lower than that of low VEGF expressors (78%) ($P = 0.01$).

### DISCUSSION

We studied VEGF expression in 60 patients with HNSCCs. High VEGF expressors by Western blot were associated with the progression of lymph-node spread, which were correlated with poor DFS.

We measured the level of VEGF protein in tumour tissue compared with adjacent normal tissue by Western blot, confirmed tumour cells expressing VEGF by immunohistochemistry, and compared the result of Western blot with that of RT-PCR or IHC. These methods need a small number of specimens, and detecting both mRNA and protein levels by the different materials and methods could guarantee the accuracy of the results. Because VEGF mRNA and proteins are being produced, transported, and degradated in situ, a single method such as measuring only mRNA or protein may fail to detect its production. Although the cut-off point between high and low VEGF expressors is optional in this

 © 2000 Cancer Research Campaign

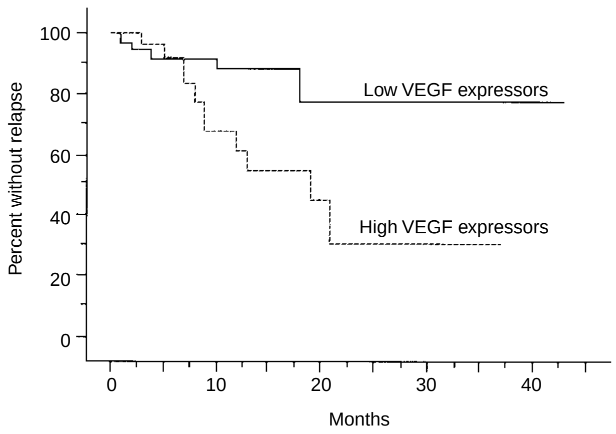

**Figure 3** The Kaplan–Meier curve for disease-free survival (DFS) showed the 2-year DFS rates of vascular endothelial growth factor (VEGF) high expressors and VEGF low expressors. The 2-year DFS rate of VEGF high expressors (30%) was significantly lower than that of VEGF low expressors (78%) ($P$ = 0.0008)

analysis of Western blot, it clearly divided into high VEGF expressors and low VEGF expressors. The result of Western blot analysis significantly correlated with that of RT-PCR according to this cut-off point criterion. Twenty-two patients out of 26 high VEGF expressors by Western blot showed two isoforms of transcripts by RT-PCR. This suggests that VEGF protein may come from newly synthesized mRNA, and not from the proteolytic cleavage of precursor proteins which were transported from other places. However, four patients showed high VEGF protein expression without VEGF mRNA expression. This discrepancy may be due to the sensitivity of antibody and sampling errors. The mRNA and the protein may be examined in different tumour areas where hypoxic normal tissue or activated macrophages secreting VEGF are contained. The detected two isoforms of transcripts showed the shorter secreted types which had biological activity. All high VEGF expressors showed VEGF-positive reactivity in immunostaining. Although fibroblasts, endothelial cells and tumour infiltrating cells were also positively stained, their contributions were minor compared to the tumour cells.

We found high VEGF expressors in 43% in HNSCC. The varied expression may depend on the differences in the source of the specimen, the method of analysis and the organ specificity. The immunohistochemical analysis revealed the positivity in a wide range, because of the differences in the criteria. The incidence of high VEGF expressors was greater in renal (Takahashi et al, 1994) or lung (Fontanini et al, 1997) carcinomas than in oesophageal (Inoue et al, 1997; Shimada et al, 1999) or gastric (Maeda et al, 1999; Saito et al, 1999) carcinomas. VEGF is generally expressed in malignant tumours, and is rarely expressed in benign tumours or normal tissues except for alveolar cells in the lung, podocytes and mesangium cells in the glomerulus, and cortical cells in the adrenal gland (Brown et al, 1992; 1993). This may suggest that the tumours originating from these organs are more likely to express VEGF than other organs. HNSCCs include heterogeneous carcinomas and could have different biological activities in each location and histology. In fact, maxillary squamous cell carcinomas exhibited the low incidence of high VEGF expressors.

The process of metastasis may take place in two stages: prevascular and vascular (Weidner et al, 1991). In the prevascular stage, marginal tumour cells locally invade host stroma surrounding the primary tumour without vascular invasion, which is associated with limited growth. In the vascular phase, tumour cells enter blood vessels or lymphatic vessels, which is associated with rapid growth or metastasis (Macchiarini et al, 1992). Although neovascularization is necessary to become the vascular phase, it is controlled by the balance of positive and negative regulators. VEGF does not stimulate the growth of tumour cells directly, but leads to the growth and increase of permeability of endothelial cells. VEGF serves to induce neovascularization around tumour cells and promotes extravasation of plasma fibrinogen, leading to the alteration of the tumour extracellular matrix (Eisma et al, 1997) and promoting metastasis by breaking down the extracellular matrix in the tumour microenvironment. These may be an important role of VEGF on tumour cells.

We found that VEGF expression was associated with lymph-node status. The previous studies in HNSCC (Eisma et al, 1997; Moriyama et al, 1997; Sauter et al, 1999) revealed that VEGF expression was concerned with tumour aggressiveness. Sauter et al (1999) documented that VEGF staining was found in the majority of advanced primary SCCs and lymph-node metastases, whereas dysplasia, carcinoma in situ, or early SCCs did not show intense immunostaining. However, some studies reported that there was no association of VEGF expression with tumour size or lymph-node status in HNSCC (Salven et al, 1997; Maeda et al, 1998; Neuchrist et al, 1999). Our study supported Sauter's results that the higher VEGF expressors were associated with lymph-node metastasis. Regional lymph-node spread is less frequently seen from tumours of the maxillary sinus clinically, which may be attributed to the low incidence of high VEGF expressors. Some studies of the microvessel density (Gasparini et al, 1993; Williams et al, 1994; Murray et al, 1997; Sauter et al, 1999) revealed that tumour microvessel density was correlated with tumour progression and recurrence. Williams et al (1994) reported that patients with > 4 mm of tumour depth and > 10% Factor VIII tumour staining had a 100% rate of recurrence, and that only angiogenesis was found to be an independent predictor of nodal metastasis, while others (Dray et al, 1995; Zätterström et al, 1995; Moriyama et al, 1997; Neuchrist et al, 1999) failed to find any association. This inconsistency may depend on: the small number of patients analysed; the heterogeneous group of tumours with different primary sites and of different stages; the different areas of each histologic sections (i.e. marginal or central of the tumour); the wide range of immunohistochemical positivity.

Multivariate analysis revealed VEGF expression and stage grouping were independent predictors for DFS, however there is controversy whether VEGF expression influences the prognosis. The 2-year DFS of high VEGF expressors (30%) was significantly lower than that of low VEGF expressors (78%). Although this study was limited to a small number of patients and a short duration of follow-up, it suggests that high VEGF expressors may need further adjunctive therapy to control lymph-node metastasis. VEGF expression may help us: to make the decision of prophylactive neck dissection; to select the patients to take the anti-angiogenetic therapy; and to confirm the pharmacological effect of anti-angiogenetic drugs. Future studies need to investigate the relationship between VEGF expression and recurrence or metastasis in a large number of patients with HNSCCs.

© 2000 Cancer Research Campaign

In summary VEGF was highly expressed in 43% in HNSCCs. High VEGF expressors were significantly correlated with lymph-node spread, and their prognosis was significantly worse than that of low VEGF expressors.

### ACKNOWLEDGEMENTS

We would like to thank Kaoru Ishikawa and Yuko Mohri for their excellent technical support and helpful discussion. This work was supported by Grants-in-Aid for Scientific Research (C)(09671741) from the Japanese Ministry of Education, Science, Sports and Culture.

### REFERENCES

Abu-Jawdeh GM, Faix JD, Niloff J, Tognazzi K, Manseau E, Dvorak HF and Brown LF (1996) Strong expression of vascular permeability factor (vascular endothelial growth factor) and its receptors in ovarian borderline and malignant neoplasms. *Lab Invest* **74**: 1105–1115

Boocock CA, Charnock-Jones DS, Sharkey AM, McLaren J, Barker PJ, Wright KA, Twentyman PR and Smith SK (1995) Expression of vascular endothelial growth factor and its receptors flt and KDR in ovarian carcinoma. *J Natl Cancer Inst* **87**: 506–516

Bouck N, Stellmach V and Hsu SC (1996) How tumors become angiogenic. *Adv Cancer Res* **69**: 134–174

Brown LF, Asch B, Harvey VS, Buchinski B and Dvorak HF (1988) Fibrinogen influx and accumulation of cross-linked fibrin in mouse carcinomas. *Cancer Res* **48**: 1920–1925

Brown LF, Berse B, Jackman RW, Togbazzi K, Manseau E, Dvorak HF and Senger DR (1993) Increased expression of vascular permeability factor (vascular endothelial growth factor) and its receptors in kidney and bladder carcinomas. *Am J Pathol* **143**: 1255–1262

Burian M, Quint C and Neuchrist C (1999) Angiogenic factors in laryngeal carcinomas: do they have prognostic relevance? *Acta Otolaryngol* **119**: 289–292

Dray TG, Hardin NJ and Sofferman RA (1995) Angiogenesis as a prognostic marker in early head and neck cancer. *Ann Otol Rhinol Laryngol* **104**: 724–729

Eisma RJ, Spiro JD and Kreutzer DL (1997) Vascular endothelial growth factor expression in head and neck squamous cell carcinoma. *Am J Surg* **174**: 513–517

Ferrara N and Henzel WJ (1989) Pituitary follicular cells secrete a novel heparin-binding growth factor specific for vascular endothelial cells. *Biochem Biophys Res Commun* **161**: 851–858

Ferrara N, Leung DW, Cachianes G, Winer J and Henzel WJ (1991*a*) Purification and cloning of vascular endothelial growth factor secreted by pituitary folliculostellate cells. *Methods Enzymol* **198**: 391–405

Ferrara N, Houck KA, Jakeman LB, Winer J and Leung DW (1991*b*) The vascular endothelial growth factor family of polypeptides. *J Cell Biochem* **47**: 211–218

Ferrara N, Houck K, Jakeman L and Leung DW (1992) Molecular and biological properties of the vascular endothelial factor family of protein. *Endocr Rev* **13**: 18–32

Folkman J (1995) Angiogenesis in cancer, vascular, rheumatoid and other disease. *Nat Med* **1**: 27–31

Folkman J, Watson K, Ingber D and Hanahan D (1989) Induction of angiogenesis during the transition from hyperplasia to neoplasia. *Nature* **339**: 58–61

Fontanini G, Vignati S, Boldrini L, Chine S, Silvestri V, Lucchi M, Mussi A, Angeletti CA and Bevilacqua G (1997) Vascular endothelial growth factor is associated with neovascularization and influences progression of non-small cell lung carcinoma. *Clin Cancer Res* **3**: 861–865

Fontanini G, Boldrini L, Chine S, Pisaturo F, Basolo F, Calcinai A, Lucchi M, Mussi A, Angeletti CA and Bevilacqua G (1999) Expression of vascular endothelial growth factor mRNA in non-small cell lung carcinomas. *Br J Cancer* **79**: 363–369

Gasparini G, Weidner N, Maluta S, Pozza F, Boracchi P, Mezzetti M, Testolin A and Bevilacqua P (1993) Intratumoral microvessel density and p53 protein: correlation with metastasis in head-and-neck squamous-cell carcinoma. **55**: 739–744

Holmgren L, O'Reilly MS and Folkman J (1995) Dormancy of micrometastases: balanced proliferation and apoptosis in the presence of angiogenesis suppression. *Nat Med* **1**: 149–153

Ikeda N, Adachi M, Taki T, Huang C, Hashida H, Takabayashi A, Sho M, Nakajima Y, Kanehiro H, Hisanaga M, Nakano H and Miyake M (1999) Prognostic significance of angiogenesis in human pancreatic cancer. *Br J Cancer* **79**: 1553–1563

Inoue K, Ozeki Y, Suganuma T, Sugiura Y and Tanaka S (1997) Vascular endothelial growth factor expression in primary esophageal squamous cell carcinoma. Association with angiogenesis and tumor progression. *Cancer* **79**: 206–213

Ishigami SI, Arii SS, Furutani M, Niwano M, Harada T, Mizumoto M, Mori A, Onodera H and Imamura M (1998) Predictive value of vascular endothelial growth factor (VEGF) in metastasis and prognosis of human colorectal cancer. *Br J Cancer* **78**: 1379–1384

Itakura J, Ishiwata T, Friess H, Fujii H, Matsumoto Y, Büchler MW and Korc M (1997) Enhanced expression of vascular endothelial growth factor in human pancreatic cancer correlates with local disease progression. *Clin Cancer Res* **3**: 1309–316

Keck PJ, Hauser SD, Krivi G, Sanzo K, Warren T, Feder J and Connolly DT (1989) Vascular permeability factor, an endothelial cell mitogen related to PDGF. *Science* **246**: 1309–1312

Kumar H, Heer K, Lee PWR, Duthie GS, MacDonald AW, Greenman J, Kerin MJ and Monson JR (1998) Preoperative serum vascular endothelial growth factor can predict stage in colorectal cancer. *Clin Cancer Res* **4**: 1279–1285

Leung DW, Cachianes G, Kuang WJ, Goeddel DV and Ferrara N (1989) Vascular endothelial growth factor is a secreted angiogenic mitogen. *Science* **246**: 1306–1309

Linderholm B, Tavelin B, Grankvist K and Henriksson R (1999) Does vascular endothelial growth factor (VEGF) predict local relapse and survival in radiotherapy-treated node-negative breast cancer? *Br J Cancer* **81**: 727–732

Macchiarini P, Fontanini G, Hardin MJ, Squartini F and Angeletti CA (1992) Relation of neovascularisation to metastasis of non-small-cell lung cancer. *Lancet* **340**: 145–146

Maeda K, Chung YS, Ogawa Y, Takatsuka S, Kang SM, Sawada T and Sowa M (1996) Prognostic value of vascular endothelial growth factor expression in gastric carcinoma. *Cancer* **77**: 858–863

Maeda T, Matsumura S, Hiranuma H, Jikko A, Furukawa S, Ishida T and Fuchihara H (1998) Expression of vascular endothelial growth factor in human oral squamous cell carcinoma: its association with tumour progression and p53 gene status. *J Clin Pathol* **51**: 771–775

Maeda K, Kang SM, Onoda N, Kato Y, Sawada T and Chung KH (1999) Vascular endothelial growth factor expression in preoperative biopsy specimens correlates with disease recurrence in patients with early gastric carcinoma. *Cancer* **86**: 566–571

Moriyama M, Kumagai S, Kawashiri S, Kojima K, Kakihara K and Yamamoto E (1997) Immunohistochemical study of tumour angiogenesis in oral squamous cell carcinoma. *Oral Oncol* **33**: 369–374

Murray JD, Carlson GW, McLaughlin K, Pennington M, Lynn M, DeRose PB, Williams JK and Cohen C (1997) Tumor angiogenesis as a prognostic factor in laryngeal cancer. **174**: 523–526

Neuchrist C, Quint C, Pammer A and Burian M (1999) Vascular endothelial growth factor (VEGF) and microvessel density in squamous cell carcinomas of the larynx: an immunohistochemical study. *Acta Otolaryngol* **119**: 732–738

O'Brien T, Cranston D, Fuggle S, Bicknell R and Harris AL (1995) Different angiogenic pathways characterize superficial and invasive bladder cancer. *Cancer Res* **55**: 510–513

O'Byrne KJ, Dobbs N, Propper D, Smith K and Harris AL (1999) Vascular endothelial growth factor platelet counts, and prognosis in renal cancer. *Lancet* **353**: 1494–1495

Olson TA, Mohanraj D, Carson LF and Ramarkrishnan S (1994) Vascular permeability factor gene expression in normal and neoplastic ovaries. *Cancer Res* **54**: 276–280

Paley PJ, Staskus KA, Gebhard K, Mohanraj D, Twiggs LB, Carson LF and Ramakrishnan S (1997) Vascular endothelial growth factor expression in early stage ovarian carcinoma. *Cancer* **80**: 98–106

Saito H, Tsujitani S, Kondo A, Ikeguchi M, Maeta M and Kaibara N (1999) Expression of vascular endothelial growth factor correlates with hematogenous recurrence in gastric carcinoma. *Surgery* **125**: 195–201

Salven P, Heikkilä P, Anttonen A, Kajanti M and Joensuu H (1997) Vascular endothelial growth factor in squamous cell head and neck carcinoma: Expression and prognostic significance. *Mod Pathol* **10**: 1128–1133

Sauter ER, Nesbit M, Watson JC, Klein-Szanto A, Litwin S and Herlyn M (1999) Vascular endothelial growth factor is a marker of tumor invasion and metastasis in squamous cell carcinomas of the head and neck. *Clin Cancer Res* **5**: 775–782

Scott PA, Smith K, Poulsom R, De Benedetti A, Bicknell R and Harris AL (1998) Differential expression of vascular endothelial growth factor mRNA vs protein

© 2000 Cancer Research Campaign isoform expression in human breast cancer and relationship to elF-4E. *Br J Cancer* **77**: 2120–2128

Senger DR, Galli SJ, Dvorak AM, Perruzzi CA, Harvey VS and Dvorak HF (1983) Tumor cells secrete a vascular permeability factor that promotes accumulation of ascites fluid. *Science* **219**: 983–985

Shibusa T, Shijubo N and Abe S (1998) Tumor angiogenesis and vascular endothelial growth factor expression in stage I lung adenocarcinoma. *Clin Cancer Res* **4**: 1483–1487

Shimada Y, Imamura M, Watanabe G, Uchida S, Harada H, Makino T and Kano M (1999) Prognostic factors of oesophageal squamous cell carcinoma from the perspective of molecular biology. *Br J Cancer* **80**: 1281–1288

Sobin LH and Witterkind Ch. (Eds) (1997) Head and neck tumours. In *International Union Against Cancer. TNM classification of malignant tumours*, 5th edn. pp 17–50. Wiley-Liss: New York

Suzuki K, Hayashi N, Miyamoto Y, Yamamoto M, Ohkawa K, Ito Y, Sasaki Y, Yamaguchi Y, Nakase H, Noda K, Enomoto N, Arai K, Yamada Y, Yoshihara H, Tujimura T, Kawano K, Yoshikawa K and Kamada T (1996) Expression of vascular permeability factor/vascular endothelial growth factor in human hepatocellular carcinoma. *Cancer Res* **56**: 3001–3009

Takahashi A, Sasaki H, Kim SJ, Tobisu K, Kakizoe T, Tsukamoto T, Kumamoto Y, Sugimura T and Terada M (1994) Markedly increased amount of messenger RNA for vascular endothelial growth factor and placenta growth factor in renal cell carcinoma. *Cancer Res* **54**: 4233–4237

Takahashi Y, Kitadai Y, Bucana CD, Cleary KR and Ellis LM (1995) Expression of vascular endothelial growth factor and its receptor, KDR, correlates with vascularity, metastasis, and proliferation of human colon cancer. *Cancer Res* **55**: 3964–3968

Takahashi Y, Tucker SL, Kitadai Y, Koura AN, Bucana CD, Cleary KR and Ellis LM (1997) Vessel counts and expression of vascular endothelial growth factor as prognostic factors in node-negative colon cancer. *Arch Surg* **132**: 541–546

Takano S, Yoshii Y, Kondo S, Suzuki H, Maruno T, Shirai S and Nose T (1996) Concentration of vascular endothelial growth factor in the serum and tumor tissue of brain tumor patients. *Cancer Res* **56**: 2185–2190

Toi M, Hoshina S, Takayanagi T and Tominaga T (1994) Association of vascular endothelial growth factor expression with tumor angiogenesis and with early relapse in primary breast cancer. *Jpn J Cancer Res* **85**: 1045–1049

Weidner N, Semple JP, Welch WR and Folkman J (1991) Tumor angiogenesis and metastasis: Correlation in invasive breast carcinoma. *New Engl J Med* **324**: 1–8

Weindel K, Marmé D and Weich HA (1992) AIDS-associated Kaposi's sarcoma cells in culture express vascular endothelial growth factor. *Biochem Biophys Res Commun* **183**: 1167–1174

Williams JK, Carlson GW, Cohen C, Derose PB, Hunter S and Jurkiewicz MJ (1994) Tumor angiogenesis as a prognostic factor in oral cavity tumors. *Am J Surg* **168**: 373–380

Yoshiji H, Gomez DE, Shibuya M and Thorgeirsson UP (1996) Expression of vascular endothelial growth factor, its receptor, and other angiogenic factors in human breast cancer. *Cancer Res* **56**: 2013–2016

Zätterström UK, Brun E, Willén R, Kjellén E and Wennerberg J (1995) Tumor angiogenesis and prognosis in squamous cell carcinoma of the head and neck. *Head Neck* **17**: 312–331

*© 2000 Cancer Research Campaign* 
